# Effect of Particle Migration on the Stress Field in Microfluidic Flows of Blood Analog Fluids at High Reynolds Numbers

**DOI:** 10.3390/mi14081494

**Published:** 2023-07-25

**Authors:** Finn Knüppel, Ang Sun, Frank-Hendrik Wurm, Jeanette Hussong, Benjamin Torner

**Affiliations:** 1 Institute of Turbomachinery, Faculty for Mechanical Engineering and Ship Design, University of Rostock, 18055 Rostock, Germany; finn.knueppel@uni-rostock.de (F.K.); hendrik.wurm@uni-rostock.de (F.-H.W.); 2 Institute for Fluid Mechanics and Aerodynamics, Technical University of Darmstadt, 64287 Darmstadt, Germany; sun@sla.tu-darmstadt.de (A.S.); hussong@sla.tu-darmstadt.de (J.H.)

**Keywords:** cell-free layer, particulate blood analog fluid, particle-laden flows, wall shear stress, astigmatism particle tracking velocimetry, apparent viscosity, Fåhræus–Lindqvist effect, microchannels, ventricular assist devices, gap flow

## Abstract

In the present paper, we investigate how the reductions in shear stresses and pressure losses in microfluidic gaps are directly linked to the local characteristics of cell-free layers (CFLs) at channel Reynolds numbers relevant to ventricular assist device (VAD) applications. For this, detailed studies of local particle distributions of a particulate blood analog fluid are combined with wall shear stress and pressure loss measurements in two complementary set-ups with identical flow geometry, bulk Reynolds numbers and particle Reynolds numbers. For all investigated particle volume fractions of up to 5%, reductions in the stress and pressure loss were measured in comparison to a flow of an equivalent homogeneous fluid (without particles). We could explain this due to the formation of a CFL ranging from 10 to 20 μm. Variations in the channel Reynolds number between *Re* = 50 and 150 did not lead to measurable changes in CFL heights or stress reductions for all investigated particle volume fractions. These measurements were used to describe the complete chain of how CFL formation leads to a stress reduction, which reduces the apparent viscosity of the suspension and results in the Fåhræus–Lindqvist effect. This chain of causes was investigated for the first time for flows with high Reynolds numbers (Re∼100), representing a flow regime which can be found in the narrow gaps of a VAD.

## 1. Introduction

**Figure 1:**
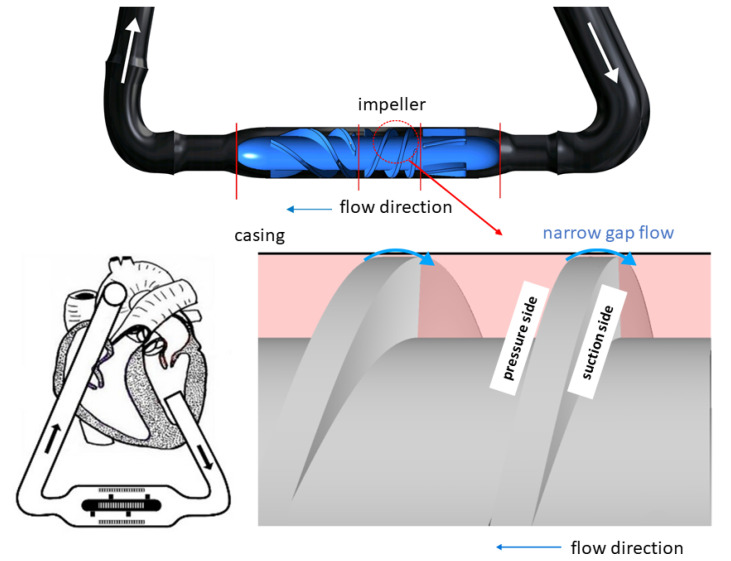
(**Left**): Axial VAD and schematic implantation situation. (**Right**): Illustration of the gap flow through the narrow gap between impeller and casing.

Around 64 million people worldwide suffer from heart failure [1]. In the case of a terminal heart failure, the heart’s performance is weakened to a degree that it can no longer supply the body with a sufficient blood flow, and a heart donation is needed as the gold standard for treatment. The 2022 report from the *Newsletter Transplant* with data from the Global Observatory on Donation and Transplantation (GODT) shows that 8409 hearts were transplanted in 2021 compared to 21,935 patients on the waiting list [2]. This urges the development of technical solutions, such as implantable ventricular assist devices (VADs). A VAD supports a weakened heart by increasing the blood pressure to overcome vessel resistance and generate a sufficient blood flow. The majority of VADs are designed as axial or radial turbomachines [3]. To generate the blood flow, the impeller rotates with several thousand revolutions per minute. These high rotational speeds lead to supraphysiological stresses $\tau_{ij}$, which are highest in the narrow VAD gaps. These narrow gaps are located in VADs, for example, in the bearing region [4], in the tip region of an axial impeller, and in the side chambers of radial impellers [5]. For example, Figure 1 shows the narrow gap in the tip region of an axial VAD, which is only one hundred micrometers in height [6,7]. Since supraphysiological stresses of several hundred Pascals occur and act on the blood cells in this gap, this region is of great interest for flow optimization purposes to increase the device’s hemocompatibility. However, little experimental research has been performed to understand the actual blood flow in narrow VAD gaps so far [4,8].

**Figure 2:**
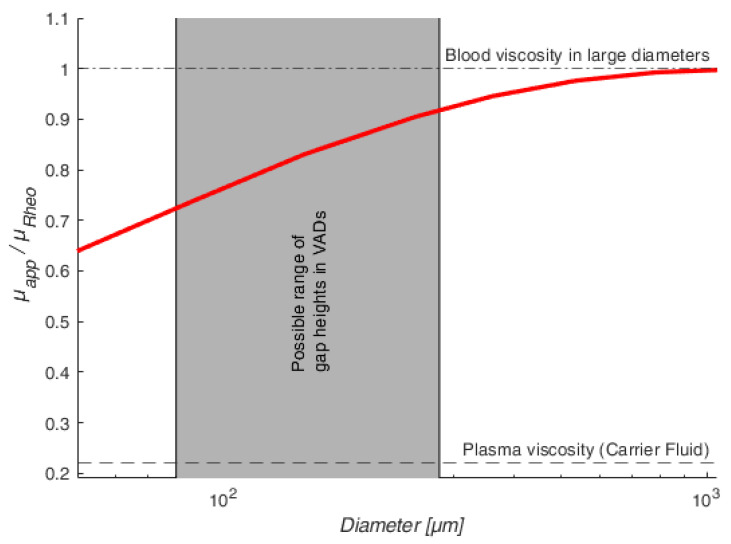
An illustration of the Fåhræus–Lindqvist effect showing the ratio of apparent viscosity $\mu_{app}$ to the bulk viscosity $\mu_{rheo}$ for ϕ=45%. This figure was built based on the data of ref. [19].

Nonetheless, the understanding of the VAD gap flow is of great importance because it is known from the literature that red blood cells (RBCs) tend to migrate and separate from plasma in flows of similar geometric dimensions [9,10,11,12,13,14]. This results in the Fåhræus–Lindqvist effect. In 1931, Fåhræus and Lindqvist made the groundbreaking discovery that the apparent viscosity $\mu_{app}$ of blood decreases when the blood flows through geometries (e.g., vessels) in the micrometer range. This viscosity decrease in microgeometries can be seen in Figure 2, where $\mu_{app}$ is compared to the bulk viscosity $\mu_{Rheo}$, which is the blood viscosity determined, e.g., by a rheometer [15]. To understand this reduction in viscosity, we need to further analyse the general reason for the Fåhræus–Lindqvist effect.

The effect originates from the flow forces acting on RBCs, which lead to a migration of the RBCs towards the center of the microgeometry and to a formation of a cell-free layer (CFL) [16]. Due to this migration, only low-viscosity plasma is present in the CFL. CFL formation reduces the shear stresses $\tau_{ij}$ in the near-wall region due to the presence of just low-viscosity plasma in the CFL, see Equation (1) (e.g., defined in ref. [17]). This reduction in shear stresses also reduces the pressure losses in the microgeometry. Fåhræus and Lindqvist accounted for this pressure reduction by introducing an apparent viscosity $\mu_{app}$, which was calculated by Poiseuille’s law, see Equation (2) (e.g., defined in ref. [18]). This equation applies to a laminar flow through a pipe of length *L*, radius *r* and flow rate *Q*, where $\Delta p_{meas}$ indicates the measured pressure loss through the microgeometry.

(1) $$\tau_{ij}{|}_{CFL}=\mu_{plasma}\cdot \left(\frac{\partial u_{i}}{\partial x_{j}}+\frac{\partial u_{j}}{\partial x_{i}}\right)$$

(2) $$\mu_{app}=\frac{Q\cdot 8\cdot \Delta p_{meas}\cdot L}{\pi \cdot r^{4}}$$

Transferring this knowledge to the VAD gap flow, VADs also have gap heights in the order of a few hundred micrometers, indicated in Figure 2. Here, cell migration effects might happen, leading to the Fåhræus–Lindqvist effect [8]. This means that the stresses in VAD gaps could also be reduced due to CFL formation, resulting in smaller supraphysiological stresses acting on the RBCs than has been assumed so far.

Therefore, it seems important to understand and characterize the migration effects in flow conditions comparable to VAD gap flows.

Nonetheless, the current flow investigations on cell migration, CFL formation and the Fåhræus–Lindqvist effect in the literature [9,10,11,12,13,14] are restricted only to physiological flow conditions, represented by small Reynolds numbers. The Reynolds numbers are in the order of $Re=c_{b}\cdot d/\mu_{rheo}∼1$, with $c_{b}$ as the bulk velocity and Re=6 as the largest value found in the literature [9].

If an equivalent Reynolds number is formed in a VAD gap flow, assuming that the diameter *d* is built from two times the gap height, a Reynolds number in the range of Re∼150 is obtained. These technically relevant Reynolds numbers are 25 times higher than the physiologically relevant Reynolds numbers of the literature experiments.

Since experiments in this Reynolds number range do not exist, the current paper aims to fundamentally analyse the CFL characteristics and basically understand the cell migration effects at high Reynolds numbers. Therefore, we have conducted experimental studies with particulate blood analog fluids (pBAFs). pBAFs are liquids with similar flow properties (density and viscosity) and corresponding flow behavior (Newtonian and non-Newtonian) to blood, in which particles are suspended that are intended to mimic the cells (in size, shape, flow behavior, etc.). The use of pBAFs offers important advantages over the use of real blood, such as better handling [20], less agglomeration [21] and, most importantly, better optical accessibility [21,22] in the particle-laden flow. By using optical-accessible pBAFs in the present study, we combine optical studies of the particle migration with a quantification of the associated stress reduction in a microgeometry to quantify the Fåhræus–Lindqvist effect at high Reynolds numbers for the first time.

For this purpose, the particle-laden flow in straight microchannels of 150μm height is studied with pBAFs at particle volume fractions of ϕ=(0.006%,3%,5%) and channel Reynolds numbers between $Re_{ch}$ = 50 and 150. Overall, this study aims to investigate the following focal points:

- The main focus is the general study of particle migration and CFL formation of pBAFs in microfluidic systems at high Reynolds numbers (Re∼100). This Reynolds number range has not been studied in the literature before, but is highly interesting since similar Re can occur in narrow gaps of VADs.
- By performing optical measurements using astigmatism particle tracking velocimetry (APTV), we measure the CFL height and determine the particle distribution in the microchannel flow. With this distribution, an inhomogeneous viscosity distribution $\mu_{loc}=f\left(h\right)$ in the pBAF flow can be determined.
- Direct measurements of wall shear stress (WSS) in the pBAF flow allow the assessment of shear stress changes due to CFL formation. For this purpose, the pBAF flow is compared to a flow of an equivalent homogeneous blood analog fluid (hBAF) with the same density ρ and corresponding bulk viscosity $\mu_{rheo}$. With these measurements, we are able to combine the optically determined results (CFL heights and viscosity distributions) with their effects on the flow (stress reduction).
- In the final step, pressure loss measurements across the microchannel are used to determine the extent to which CFL formation and shear stress reduction lead to a reduction in pressure losses in the channel. Based on these results, an apparent viscosity $\mu_{app}$ is determined for the pBAF to quantify the Fåhræus–Lindqvist effect for the investigated parameter range. To our knowledge, this is the first time that the complete chain leading to this effect has been described for a pBAF flow as well as for high Reynolds number flows.

Addressing all of these issues provides the basis for a deeper understanding of real flow conditions in narrow VAD gaps at high Reynolds numbers, where particle/cell migration might greatly affect the flow.

## 2. Materials and Methods

In this section, we explain how we studied the particle migration, CFL foration and the Fåhræus–Lindqvist effect in the flow of the particulate blood analog fluid (pBAF). For this purpose, Section 2.1 explains the microchannel geometries and the flow conditions of the microchannel experiments. Subsequently, the fluid properties of the pBAFs are characterized by different particle volume fractions in Section 2.2 and Section 2.3. Laser optical experiments were performed with these fluids to identify particle migration and CFL formation, which is described in Section 2.4. In Section 2.5, we explain how we determined the shear stresses and pressure losses in the channel flow with the pBAF using mechanical measurement techniques.

Our hypothesis is that a reduction in stresses and pressure losses in the channel occurs through CFL foration in the pBAF. In order to quantify this reduction, we compared the results of the experimental pBAF flow with simulation results of a (particle-free) homogeneous fluid, with the same density ρ and bulk viscosity $\mu_{rheo}$. The set-up for these simulations is explained in Section 2.6. In the last Section 2.7, we explain the methodology of how we determine a local viscosity distribution $\mu_{loc}=f\left(h\right)$ and an apparent viscosity $\mu_{app}$ from all results to identify the Fåhræus–Lindqvist effect in the pBAF.

### 2.1. Microchannel Geometries for the Experimental Analysis of the Particle-Laden Flow

**Figure 3:**
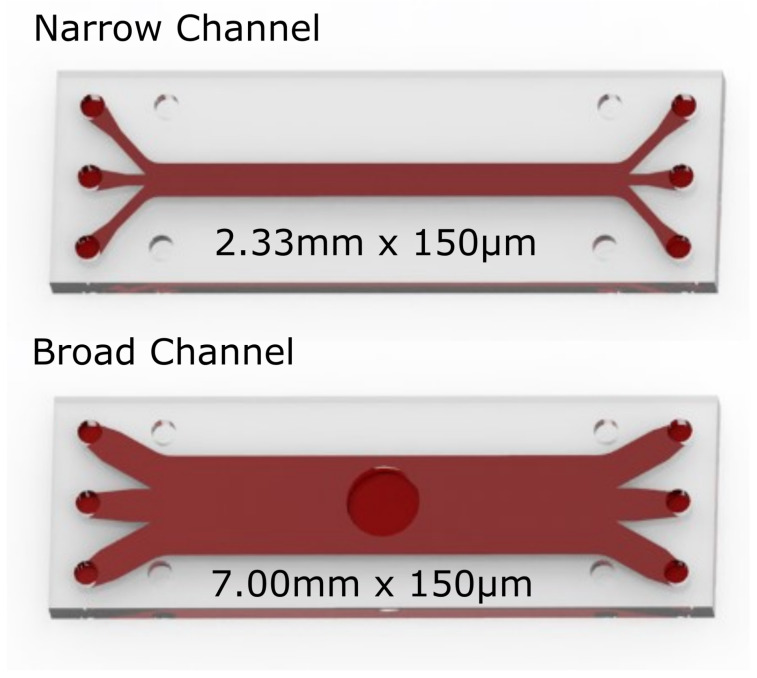
The two microchannels used in this study. The narrow channel is intended for the laser optical as well as for the pressure loss measurements. The broad channel contains a hole in the middle of the channel and is designed for wall shear stress measurements.

The particle-laden flows in the pBAFs were analysed in straight microchannels. These were made of borosilicate glass by *Micronit Microfluidics BV (Enschede, NL)* and were fabricated using an isotropic wet etching technique. Two channel variants were designed and used to integrate the various measuring methods. These channels are illustrated in Figure 3. Both channels have a height of h=150μm, which is a typical height of the flow gap in axial VADs [23].

The first channel variant is a rectangular microchannel with a length and width of 42.5mm (l) × 2.33mm (w). This first variant is called the *narrow channel* and is used for the majority of the measurements, specifically the laser optical measurements (for identification of the CFL) as well as the pressure loss measurements (for identification of the Fåhræus–Lindqvist effect) performed here. The second variant is the *broad channel*. It has dimensions of 42.5mm (l) × 7mm (w) and is used for determining the wall shear stresses (WSS) in the microchannel. The WSS are determined by a WSS sensor that is integrated directly into the duct flush with the wall, which is why a larger channel width than with the *narrow channel* was necessary. For the flush wall installation, a 5 mm wide hole was made in the *broad channel* by laser cutting on the upper wall.

The flows in both channels can be considered as plane channel flows since the aspect ratios w/h are far greater than 7 [24]. The aspect ratios for the two channels are 15.5 for the *narrow channel* and 46.6 for the *broad channel*. This is a necessary criterion to guarantee that the flow and the particle migration in both channels are not affected by the channel’s side walls.

(3) $$Re_{ch}=\rho \frac{c_{b}\cdot D_{h}}{\mu_{rheo}}$$

(4) $$Re_{p}=Re_{ch}{\left(D_{p}/h\right)}^{2}$$

Reynolds number similarities were used in order to guarantee similar flow conditions in both channels. Here, two Reynolds numbers are important for the dynamics of particulate flow, see Equations (3) and (4). The channel Reynolds number $Re_{ch}$ refers to the fluid flow (e.g., defined in ref. [25]). It is formed by the bulk (mean) velocity $c_{b}$ in the channel and the hydraulic diameter of the plane channel $D_{h}=2h$ (where *h* is the channel height). Furthermore, the fluid properties of density ρ and bulk dynamic viscosity $\mu_{rheo}$, which are measured via a rheometer, are important. The variable $\mu_{rheo}$ represents the dynamic viscosity of the suspension with homogeneous particle distribution.

Furthermore, the particle Reynolds number, $Re_{p}$, from ref. [26] indicates whether finite particle size effects play a role in the flow. It is calculated by taking the ratio between the particle diameter $D_{p}$ and the channel height *h* [27]. It is assumed that at very small particle Reynolds numbers $Re_{p}$, the particles do not disturb the underlying flow field [28]. Contrarily, when the particle Reynolds number is higher than $Re_{p}\geq 10^{-1}$, as it is in the present paper, finite size effects are encountered and disturb the underlying flow.

### 2.2. Fluids

The particulate blood analog fluids (pBAFs) used in this study consist of two fluidic systems. The fluidic systems are summarized in Table 1. In both, a carrier fluid carries spherical particles of equal size ($D_{p}=7.76\;\mu$m), corresponding to the dimensions of RBCs [29].

Firstly, a pBAF is used, which is based on a glycerol–water–ammonium thiocyanate mixture. This fluid is called a refractive-index-matched (RIM) fluid and has been successfully applied in preceding studies to study the suspension dynamics in square capillaries by means of laser optical measurements [30]. The density of the RIM fluid is adjusted to achieve a density match with the PMMA particles, thereby rendering the particles neutrally buoyant within the suspension. Crucially, by matching the refractive index of the RIM fluid with that of the PMMA particles, the turbidity of the suspension with a high particle volume fraction is diminished, resulting in an optically well-accessible suspension [31]. For the experiments, an RIM fluid with different volume fractions (ϕ=(0.006%,3%,5%)) of spherical PMMA particles ($\rho_{PMMA}=1187$ kg/m${}^{3}$) is used.

Secondly, a particulate BAF is used, which consists of water–polyethylenglycol (PEG) 200 mixtures with polystyrol (PS) particles ($\rho_{PS}=1050$ kg/m${}^{3}$). Furthermore, the PS particles were suspended in the carrier fluid at different volume fractions (ϕ=(0%,3%,5%)).

As explained above, an RIM fluid is used for the laser optical analyses due to its superior properties for optical analyses. However, it cannot be applied for the mechanical analyses with the metallic WSS sensor due to its highly corrosive behavior [32]. Instead, PEG is used for these mechanical analyses. PEG has a lower density than RIM fluid (see Table 1), with a comparable value to the density of blood [33].

The flow between both fluidic systems was equated by ensuring that the channel and particle Reynolds numbers for flow analyses at fixed volume fractions were the same. These flow analyses were performed at different channel Reynolds numbers between $Re_{ch}$ = 50 and 150. This Reynolds number range represents a flow regime of high technical relevance, but investigations of the particle migration of blood or blood analog fluids have not been investigated. In the microchannels, a constant flow rate was achieved based on the Reynolds number. A pulsating flow, as studied for some blood flows in macro-geometries in the literature [34], was not considered.

### 2.3. Fluid Properties and Rheology

**Figure 4:**
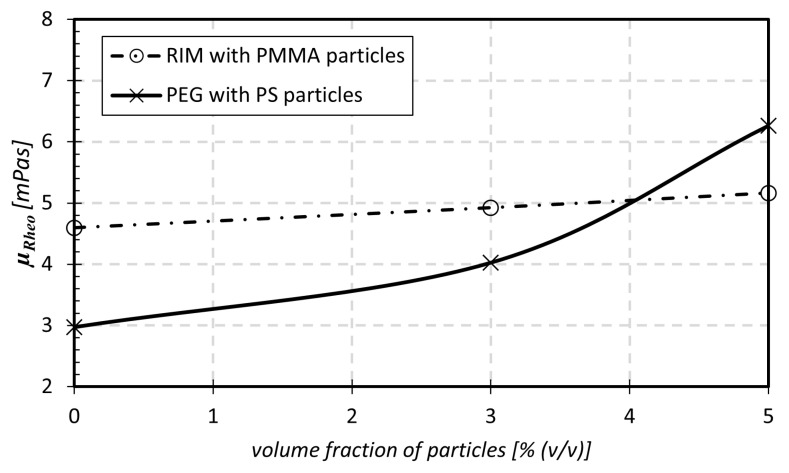
Rheological characterization of $\mu_{Rheo}$ for the two fluidic systems with different volume fractions of particles.

Density measurements and rheological characterizations of the fluidic systems were carried out by means of a Stabinger viscosimeter (falling ball viscometer, *DMA 4500, Anton Paar Group AG, Graz, Austria*) Figure 4 illustrates the rheological bulk viscosity $\mu_{Rheo}$ of PEG and the RIM fluid measured for different particle volume fractions. The fluid characteristics were measured at temperatures of $\vartheta ={(23\pm 1)\;}^{\circ }$C, which was also the temperature in the microchannel experiments. At least two replications with fresh samples were made in each measurement. Both fluidic systems are assumed to behave as Newtonian fluids. Considering only a Newtonian pBAF seems sufficient for our analyses since the shear rates exceed $S_{ij}\gg 100\;1/s$ in the microchannel experiments for the applied Reynolds numbers. In relation to this, it has been shown in previous studies that blood also exhibits Newtonian behavior for these high shear rates $S_{ij}$ [35,36].

As shown in Figure 4, the fluidic system of PEG–PS shows a more pronounced increase in viscosity with increased particle volume fraction ϕ compared to the RIM–PMMA system. The reason for this behavior is an interaction of the PS particles with the hydroxyl group of PEG. This results in a dense arrangement of the molecules in bond, which increases density and viscosity of PEG–PS [37]. The results from both fluidic systems were compared by equating the Reynolds numbers $Re_{ch}$ and $Re_{p}$, Equations (3) and (4).

### 2.4. Optical Determination of the Particle Migration and CFL Formation by Means of APTV

For the assessment of particle positions in pBAFs and the evaluation of the CFL, the astigmatism particle tracking velocimetry (APTV) technique was utilized. APTV is a single-camera technique allowing to extend a planar image-based 2D-PTV approach to a 3D tracking approach with the help of astigmatic particle images. Astigmatism is introduced by placing a cylindrical lens into the optical path, causing particle images to deform depending on its out-of-plane distance to the reference planes of the optical system [38,39]. An identification of the particle distribution can be realized from the height direction (top view) by the APTV technique. As an example, a real-time image captured by the CCD camera is shown in Supplementary Material, where particle images with different aspect ratios represent particles at different heights (*h*-positions).

**Figure 5:**
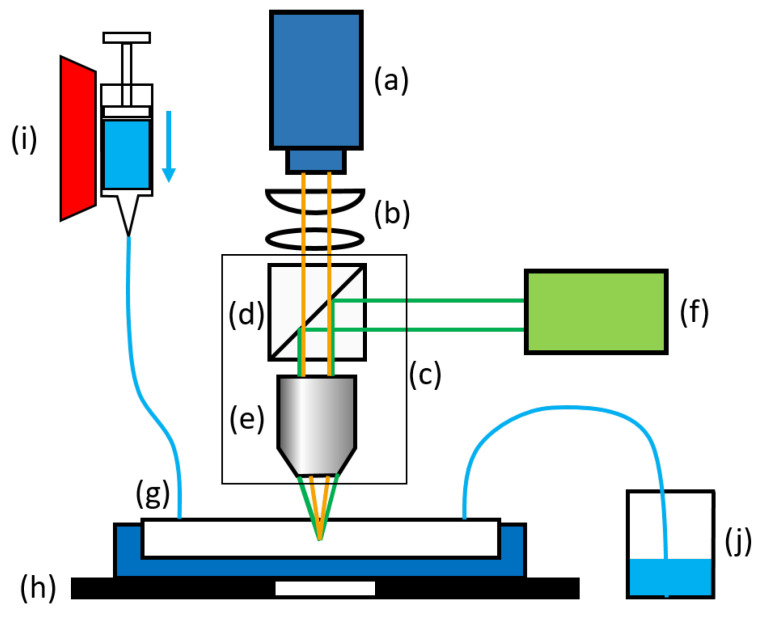
Sketch of APTV setup: (**a**) CCD camera, (**b**) cylindrical lens and field lens, (**c**) EPI-fluorescence microscope, (**d**) dichroic mirror with band-pass filters, (**e**) microscope objective, (**f**) Nd:YAG laser, (**g**) microchannel and channel holder, (**h**) XYZ-traverser, (**i**) syringe pump and syringe, (**j**) container.

For dilute particle systems, the out-of-plane position of a particle can be reconstructed from the astigmatic image shape with an accuracy that is on the order of magnitude of the particle diameter itself [39,40]. The reconstruction accuracy is implicitly related to the effective measurement volume depth, covering approximately 70μm to 85μm for the experimental system used for the present study. As shown in Figure 5, an upright epi-fluorescence microscope (*Eclipse LV100, Nikon Corporation, Minato (Tokio), Japan*) is used for the APTV measurements with a magnification of 20 and a cylindrical lens placed in front of a dual-frame CCD camera (*Imager SX 6M, LaVision GmbH, Göttingen, Germany*) of 200 mm focal length.

**Calibration.** For calibration, labeled suspension particles with a particle volume fraction of ϕ=0.006% were flushed into the microchannel and recorded two hours later after completely settling at the microchannel bottom. For this, a set of 200 images were taken at different reference planes separated for Δh=1μm in the out-of-plane direction. As the particle position relative to the channel walls is known for sedimented particles, a calibration curve can be generated to relate the particle image deformation to the distance between (in this case the known out-of-plane particle position) to the chosen reference plane of the optical system. Astigmatic images of the particles assume elliptical shapes of varying aspect ratios. Therefore, calibration curves are typically given as function of their major and minor axis length $a_{y}$ and $a_{x}$. The calibration curve an evaluation procedure allows us to reconstruct the out-of-plane particle positions of particles during a flow measurement experiment. The procedure is described in detail in work by Brockmann et al. [30]. As the current set-up shows a small hysteresis in the traversing system, the calibration procedure was extended here to account for random absolute reference plane shifts, which are assumed to be ±7μm. This was executed by evaluating the velocity profiles of the given Poiseuille flow for each flow measurement. Knowing the velocity profile and the no slip velocity at the channel walls, this velocity information can be used to correct for reference plane shifts. In this way, the out-of-plane absolute position of particle distributions can be determined to an accuracy of ±2μm.

**Flow measurements.** The same carrier liquid mixture as for the calibration was used with different volume fractions ϕ for the flow measurements. For each suspension, fluorescently labeled particles of the same size and material were added so that ϕ=0.006% particles were labeled. A constant flow rate over time was ensured through a programmable syringe pump for each Reynolds number of $Re_{ch}=50,100,150$. All astigmatic particle image recordings were illuminated with a double-pulsed laser (*Solo PIV Nd:Yag Laser System, New Wave Research Inc., Fremont, CA, USA*) and were recorded with a dual-frame CCD camera (*Imager SX 6M, LaVision GmbH, Göttingen, Germany*). Particle images assume minimal and maximal image sizes of 20–200 pixels depending on their astigmatic aberration. Double images are taken 12 mm downstream of the channel inlet at 13 Hz frequency with interframing times of 20μs, resulting in particle image displacements of 20–160 pixels. A set of 200 images were taken at each measurement plane position. Five measurement planes were chosen with a spacing of 37.5μm for each experimental parameter combination. Based on the particle image density, this resulted in more than $10^{4}$ particle images for each parameter combination that was evaluated to extract velocity profiles and density distributions over the channel height.

Due to the recording of double images, the same recording set can be used for the determination of velocity profiles, particle distributions and thus the CFL heights.

**Image processing and data analysis.** For the determination of velocities, raw images were pre-processed to enhance the signal-to-noise ratio utilizing a median filter with a kernel size of 7 px × 7 px and a bandwidth filter to filter structures of 5 to 50 pixels in diameter. In the follow-up step, particle image center points were detected in double frames. To track particle displacements, a standard in-house 2D particle tracking algorithm was applied based on a two-time-step-based nearest neighbor approach. This is also possible for cases of higher particle volume fractions, since a fixed amount of suspension particles of 0.006% was always labeled within the suspension. As all other refractive-index-matched suspension particles were not labeled, they remained invisible in fluorescent imaging, so the optical particle image density is independent of the actual particle volume fraction. The ratio of particle image spacing to particle image displacement was greater than one in the current measurements.

After a velocity was assigned to each particle, its out-of-plane position was evaluated based on its astigmatic image. For this, particle image results were plotted in the $a_{y}$−$a_{x}$ plane and a *h*-position was assigned to each particle based on the corresponding position on the calibration curve that results from the Euclidean distance approach (see also [30]).

### 2.5. Determination of the Impact of the CFL on the Flow Using Pressure Loss and Wall Shear Stress Measurements

In addition to the test rig for the laser optical APTV measurements, the same test rig was built in which pressure (losses) and wall shear stress measurements were carried out. The aim of these measurements is to capture the effect of the CFL on the near-wall stresses and pressure losses in the microchannels. Our hypothesis is that the CFL in the pBAF will reduce the high near-wall stresses in the channel, thereby decreasing the pressure loss in the whole rig compared to the flow of an equivalent, homogeneous fluid with the same density and viscosity (called homogeneous blood analog fluid, hBAF, in the following). This reduction in acting stresses due to CFL formation is detectable with our WSS sensors.

**Figure 6:**
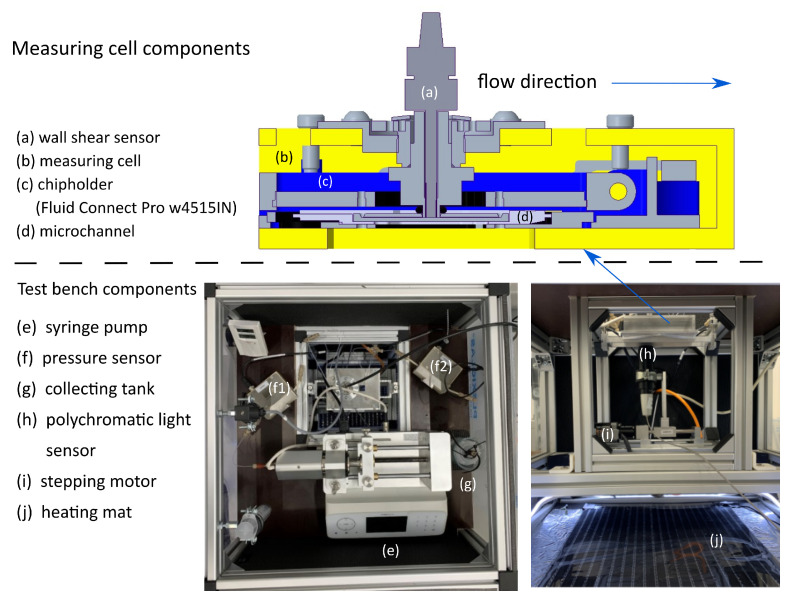
Test rig for the pressure (loss) and wall shear stress measurements in the microchannels.

The test rig is shown in Figure 6. The components are integrated into a heat chamber, where the pBAFs are heated to a temperature of $\vartheta =23{\;}^{\circ }$C by a heating mat (j). Other components include a syringe pump (e) with a 100 mL syringe (*Fusion 6000-x, Chemyx Inc., Stafford, TX, USA*), tubing connectors and a collection tank (g). The pressure drop in the rig is accounted for by two absolute pressure sensors ((f), *IS-P Tube Pressure Senors, HiTec Zang GmBH, Herzogenrath, Germany*), which are placed before (f1) and behind (f2) in the microchannel.

The core of the test rig is the measuring cell (b), in which the microchannels (d) are positioned. In the case of the *broad channel*, the wall shear stress sensor ((a), *RealShear M-Series, Lenterra Inc., Newark, NJ, USA*) is inserted into the hole at the top wall of the microchannel. The wall shear stress sensor has a swimmer at the head, which is deflected by the wall shear stress in the flow. Two optical fibers are installed in a fiber Bragg grating on the swimmer. The deflection of the swimmer changes the reflected wavelength in the grating, which is correlated with the corresponding wall shear stress. This swimmer must be integrated flush with the wall in the microchannel so that it does not actively influence the flow. For this purpose, a polychromatic light sensor (h) and a stepper motor (i) were used, with which a desired installation height of ≈1 μm could be realized.

### 2.6. Flow Simulations of the hBAF Flow

Flow simulations of the whole test rig for the mechanical analyses were performed by direct numerical simulation (DNS). These simulations were performed assuming homogeneous blood analog fluids (hBAFs). These are fluids where no particle migration effects take place and which have the same densities ρ as well as bulk viscosities $\mu_{rheo}$ as shown in Table 1 and Figure 4. The motivation for these flow simulations is to compare the effect of particle migration and CFL formation on the stress field and pressure losses in the pBAF flow to an equivalent hBAF flow where migration effects do not exist.

**Figure 7:**
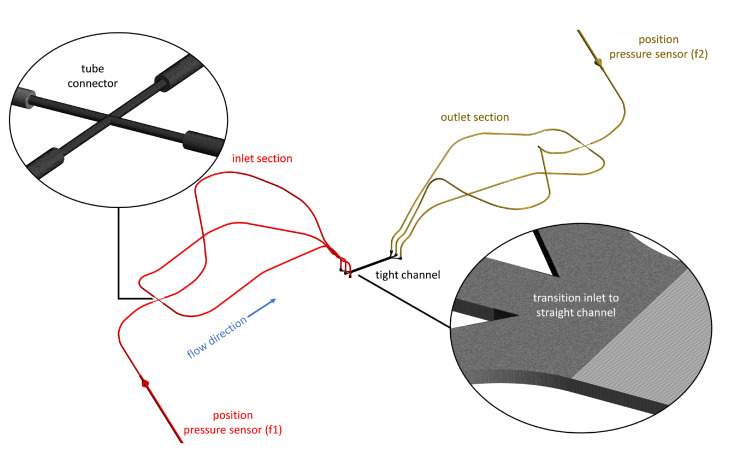
Computational domain of the test rig for the mechanical analyses with the narrow channel. Two sections of the grid are shown.

The computational domain of the simulation with the used computational grid is shown in Figure 7. The domain is a virtual representation of the test rig (both channel variants were considered) shown in Figure 6 from the inlet pressure sensor (f1) to the outlet pressure sensor (f2) via the microchannel. The grid contains ≈34 million nodes for both channel variants with angles $\geq 32^{\circ }$ and aspect ratios ≤27. The whole periphery and inlet section of the microchannel were meshed using tetrahedral elements (with five prism layers near the walls), while the straight section of the channel was meshed using hexahedral elements.

The simulations were performed by solving the Navier–Stokes equations in ANSYS CFX 2022R2 (Ansys Inc., Pittsburgh, PA, USA) with a finite volume method. A second-order spatial discretization scheme was used and a time-independent flow situation was assumed. All walls were considered to be hydraulically smooth; a laminar Hagen–Poiseuille velocity profile, corresponding to $Re_{ch}$, was assumed at the inlet; and a constant zero pressure was set at the outlet of the domain.

Convergence was reached in the simulation when the root mean square residuals dropped below $10^{-4}$ and all monitored variables (pressure loss via the test rig, wall shear stress at the channel walls, flow rates in the tubes) showed a constant progression.

### 2.7. Determination of the Global, Apparent Viscosity $\mu_{app}$ and Local Viscosity $\mu_{loc}=f\left(h\right)$

**Figure 8:**
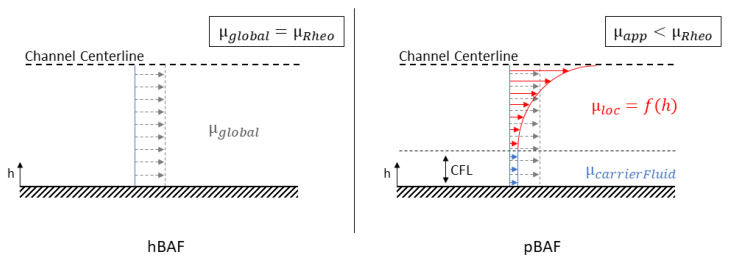
Hypothesized viscosity relations in the microchannel flows. (**Left**): Constant (global and local) bulk viscosity $\mu_{Rheo}$ of the homogeneous BAF (hBAF). (**Right**): CFL and hypothesized local viscosity distribution in the particulate BAF (pBAF) leading to a global, apparent viscosity $\mu_{app}$ smaller than $\mu_{Rheo}$.

With the help of all experiments and flow simulations, the local viscosity distribution $\mu_{loc}=f\left(h\right)$ and the global, apparent viscosity $\mu_{app}$ were determined in the microchannels at different volume fractions and Reynolds numbers. We expect that the acting forces will lead to a particle migration in the pBAF with CFL formation at the wall. This particle migration will affect the height-dependent local viscosity, with a viscosity value of the carrier fluid in the CFL zone, see the right subfigure in Figure 8. The particle distribution along the channel height is measured by the APTV measurement technique. With this distribution, the local viscosities can be calculated by using the viscosity curves proposed by Einstein and Roscoe [41]. Equation (5) from ref. [41] couples the volume fraction of the particles at a specific height *h* with the associated, local viscosity $\mu_{loc}$ at this height *h*.

(5) $$\mu_{loc}\left(h\right)=\mu_{rheo,0\%}{\left(1-3.5\cdot \phi_{loc}\left(h\right)\right)}^{-2.5}$$

The acting stresses in the flow will be altered due to this non-uniform local viscosity distribution. In particular, the highest stresses near the wall should be reduced by CFL formation due to the sole presence of the carrier fluid at the wall (see the schematic illustration in Figure 8). This reduction in stresses with the pBAFs is detected by experimentally determining the WSS in the CFL of the pBAF in the first step. In a second step, this WSS value is then compared to the WSS of the equivalent hBAF from the flow simulations, where no particle migration and CFL effects exist.

The wall shear stresses were transformed into a friction coefficient $c_{f}$ (Equation (6), ref. [42]) to compare between different volume fractions ϕ at one Reynolds number. This is necessary since it is seen from Equation (3) and Figure 4 that $\mu_{rheo}$ and $c_{b}$ change between the volume fractions at one Reynolds number. Thus, the absolute values of WSS differ also with ϕ and can only be compared by transforming them to the dimensionless quantity $c_{f}$.

In order to also compare between different feasible Reynolds numbers, $c_{f}$ is further normalized with the $c_{f}$ value of the carrier fluid (ϕ=0%) for the results. Thus, both volume-fraction-dependent and Reynolds-number-dependent trends in the stress reduction can be identified.

(6) $$c_{f}=\frac{\tau_{w}}{\rho /2\cdot c_{b}^{2}}$$

Since the stresses are connected with dissipative losses [43], the pressure loss in the channel will be reduced in the pBAF flow compared to an equivalent hBAF with the same density ρ and viscosity $\mu_{Rheo}$. This global effect of CFL formation on the pressure losses can be measured by the pressure sensors in the test rig. By comparing the pressure losses between the pBAF flow from the experiments and the hBAF flow from the simulations (both with the same ρ and $\mu_{Rheo}$), a statement can be made as to which extent the particle migration leads to reduced losses in the pBAF flow. Furthermore, the pressure losses were analysed in relation to the dimensionless pressure coefficient $c_{p}$ (Equation (7), ref. [42]) to better compare the results between volume fractions and Reynolds numbers.

(7) $$c_{p}=\frac{p_{f_{2}}-p_{f_{1}}}{\rho /2\cdot c_{b}^{2}}$$

In the style of the groundbreaking blood flow experiments of Fåhræus and Lindqvist [15], the global apparent viscosity $\mu_{app}$ was calculated from all results as a last step. The global apparent viscosity $\mu_{app}$ is a representative of the loss reduction in the channel due to CFL formation. Following Fåhræus and Lindqvist, who defined the apparent viscosity by Pouiseulle’s law (Equation (2)) from measured pressure losses in capillary tubes with a blood flow, it is defined as the viscosity which is possessed by a homogeneous fluid causing the same, reduced pressure losses as the equivalent pBAF in the microchannel.

To determine this apparent viscosity, we reduced the viscosity of the hBAF flow simulations until the corresponding $c_{p}$ values of the pBAF flows from the experiments were reached. These reduced viscosities were then defined as the global, apparent viscosities $\mu_{app}$ of the pBAF.

## 3. Results and Discussion

The results of astigmatic particle tracking velocimetry, wall shear stress and pressure loss measurements are analysed in the following sections to gain a complete picture of local, particulate flow properties, such as inhomogeneous particle concentrations, and understand their impact on global flow properties such as the shear stresses and pressure losses.

### 3.1. Characterization of the Particle Distributions in the pBAF Flows

**Figure 9:**
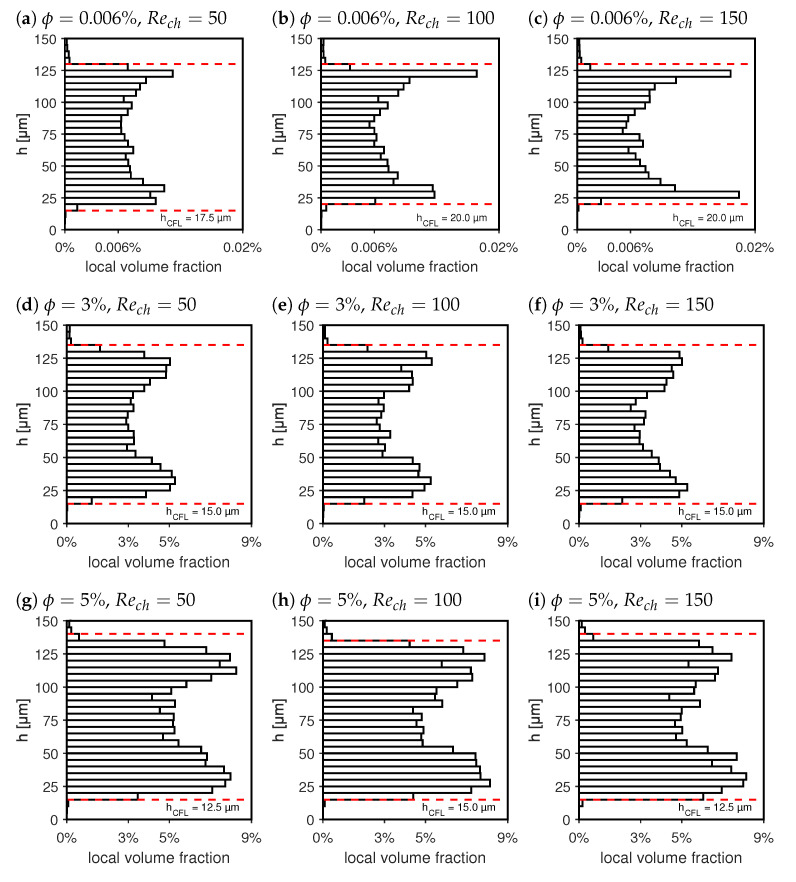
Distribution of the local volume fraction $\phi_{loc}$ for suspensions of ϕ=0.006%, 3%, 5%. The red dashed lines are the boundaries of the CFL, which is defined as the local particle volume fraction below 1/10th of its overall mean value.

Particle distributions for different channel Reynolds numbers $Re_{ch}=50,100,150$ and different mean particle volume fractions of ϕ=(0.006%,3%,5%) are plotted in Figure 9a–i. Here, the results are displayed as box plots, showing 30 median local particle volume fraction values over the channel of h=150μm. By clustering local fractions into Δh=5μm regions, a sufficient sample size is ensured for each displayed result. Clearly, an inhomogeneous particle distribution can be observed in all evaluated conditions, as expected due to the lateral migration of particles under the influence of inertial migration effects. For the depicted particle size of 7.76μm and channel height of 150μm, the particle Reynolds numbers that correspond to the displayed cases read $Re_{p}=0.13,0.25,0.38$. Within this range of particle Reynolds numbers, inertial migration effects are anticipated [27].

In Figure 9a–c, a dilute suspension flow is investigated for increasing channel Reynolds numbers. According to Zhang et al., Amini et al. and others [44,45], two inertial migration forces dominate particle migration in stationary, dilute suspension flows of neutrally buoyant particle systems. These are the wall lift force pointing away from the walls and a counteracting force, the so-called shear-gradient force [46,47,48], pointing towards the wall. At a certain off-center position, these two forces balance, leading to a so-called Pseudo Segre Silberberg Annulus (PSSA), along which particles will align [49]. In Figure 9a–c, a near-wall region can be observed in which a strong depletion of particles occurs. This is the cell-free layer (CFL) and it is formed and maintained by wall lift forces acting on the particles.

To formulate a quantitative measurement for the CFL height, we will herein refer to the CFL as the near-wall region, where the local volume fraction $\phi_{loc}$ falls locally below 1/10th of the bulk volume fraction ϕ of the suspension, denoted as $\phi_{loc}\leq 0.1\cdot \phi$. These wall distances were calculated based on the box plots with median, local particle fractions computed within the field of view (x-y plane, when *h* is z-axis) over channel height increments of Δh=5μm. The resulting positions are plotted as red dashed lines in Figure 9a–i.

Between the channel center line and the CFL region, a PSSA region can be identified with increased local volume fractions. As shown in Figure 9a–c, the formation of the PSSA in dilute suspension becomes more evident at increasing channel Reynolds numbers. Overall, these variations in local volume fraction lead to a spatially varying local viscosity distribution $\mu_{loc}=f\left(h\right)$, as discussed in the following paragraphs.

In Figure 9d–f, the local volume fractions are displayed over the channel height at ϕ=3% for increasing channel Reynolds numbers. With a higher particle volume concentration, the particle interaction forces come into play. These forces lead to an additional migration effect, referred to as shear-induced migration [50]. This migration effect drives particles away from regions of strong interaction. Naturally, these are regions of high local volume fractions or regions of high shear gradients in the flow. High shear gradients facilitate particle interactions, as the increased relative velocity difference of neighboring particles increases the probability of particle collisions. Thus, shear-induced migration is expected to lead to a drift of particles away from the PSSA where maximum local volume fractions are reached.

On the one hand, particles will migrate towards the channel center, where shear rates are low or even zero along the channel center line. On the other hand, particle local volume fractions are nearly zero in the CFL, which also suggests a migration of particles towards the wall. However, here, the wall lift forces grow rapidly with decreasing wall distances. It is known from the literature that the net lift force, which is the sum of the shear gradient force and the wall lift force near the wall, scales proportionally to the relative particle–wall distance d/h as $\left(1-2d/h\right)\left(D_{p}^{6}/h^{4}\right)$, as derived by Di Carlo et al. and Asmolov et al. [51,52]. Furthermore, shear rates are much higher between PSSA and the wall, assuming a maximum at the wall itself. Overall, this ensures that the CFL is also maintained for higher volume fractions ϕ, while the overall particle volume fraction grows in the channel center region, as observed in Figure 9d–i.

The CFL height is indicated in Figure 9a–i with a red dashed line based on the criterion formulated above. When comparing distributions in Figure 9a–c,g–i for the same channel Reynolds numbers, a weak reduction in the CFL height can be observed for increased volume fractions ϕ. On the other hand, with an increase in the channel Reynolds number, no measurable changes in the CFL height were detected for the same bulk particle volume fractions. As particle concentrations over the channel height have been evaluated in steps of Δh=5μm, a change in CFL height is expected to stay within this range. In suspensions of low particle fractions, the CFL height is directly linked to the PSSA, the wall distance at which the shear gradient and wall lift force are at equilibrium such that the resulting net force equals zero. Asmolov [51] showed that for a plane channel flow, the PSSA shifts only slightly by about a relative distance of 0.042 times the channel height if the Reynolds number triples from $Re_{ch}=67$ to $Re_{ch}=200$. This corresponds to a shift of approximately 6.3μm, which is in line with our experimental observations. The reason that the PSSA position and with it the CFL height are insensitive to the channel Reynolds number originates from the fact that the wall lift force increases sharply close to the wall, such that an increase in shear gradient force has little effect on the position of the force equilibrium.

The local particle distribution directly influences the local viscosity distribution of the flow. As can be seen from Equation (1), this will also influence the near-wall stresses in the CFL and affect the pressure losses of the channel. Thus, local volume fractions in combination with local viscosity distributions help to better understand the impact on the whole flow, which is explained in Section 3.2.

**Figure 10:**
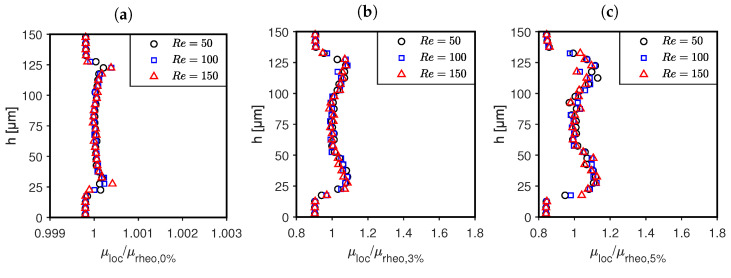
The relative viscosity distributions, where $\mu_{rheo,0\%}$, $\mu_{rheo,3\%}$ and $\mu_{rheo,5\%}$ are the rheological viscosity of the corresponding pBAF, plotted over the channel height *h* for three different Reynolds numbers for a particle volume fraction of (**a**) ϕ=0.006%, (**b**) ϕ=3% and (**c**) ϕ=5%.

The relative viscosity distribution $\mu_{loc}/\mu_{rheo}$ is plotted over the channel height *h* in Figure 10a–c. Here, it can be seen that the specific volume fraction curves fall on top of each other at different Reynolds numbers, confirming that the influence of the Reynolds number for the examined conditions has a minor effect on the particle migration dynamics. As expected, the normalized viscosity assumes values above one in regions of increased particle local volume fractions and values below one in particle-depleted regions (CFL).

The particle local volume fraction is equal to zero near the the wall in all cases. In this region, the local viscosity equals the viscosity of the pure carrier fluid and the relative viscosity $\mu_{loc}/\mu_{rheo}$ assumes values of 1.00, 0.90 and 0.85.

The local viscosity $\mu_{loc}$ and the velocity gradients $\partial u_{i}/\partial x_{j}$ in the CFL determine the corresponding wall shear stresses, see Equation (1). These are measured by means of an integrated wall shear stress sensor and are displayed in Table 2 as normalized friction coefficients. If the velocity profile over the channel height is not significantly altered from the parabolic shape of the corresponding hBAF flow, the stresses in the pBAF are reduced compared to the corresponding hBAF, as $\mu_{loc}/\mu_{rheo}$ at the wall falls below one.

This effect can be seen from the measurements with the wall shear stress sensor, shown in Table 2. Here, the wall shear stresses are presented in the form of a normalized friction coefficient $c_{f}/c_{f,0\%}$, where $c_{f}$ has been unified with the respective 0% values, i.e., the case of a flow in a pure carrier liquid. The gray area in this table represents ranges which could not be approached by the WSS sensor, since pressure values of 6 bar occurred in the *broad channel*, for which the test rig was not designed. This applies to volume fractions at 5% and Reynolds numbers above $Re_{ch}=75$. Nonetheless, from the remaining measurements, valuable insights into the WSS within the CFL can be derived.

As can be seen from the table, all results with the hBAF indicate a normalized value of one, which is expected as $c_{f}$ is a dimensionless number which should be equal for a homogeneous fluid flow at one Reynolds number. Due to the further normalization of $c_{f}$ with it fraction-specific 0% value, the value $c_{f}/c_{f,0\%}$ must also remain one for all homogeneous fluids at all Reynolds numbers.

However, this is not the case for the pBAF, where a reduction in the normalized friction coefficient is recognizable compared to the hBAF in the range of $c_{f}/\left(c_{f,0\%}\right)$ = 0.87–0.93. This is due to the aforementioned reduction in local viscosities in the CFL, which leads to a reduction in wall shear stresses in the pBAF. What is further noticeable for ϕ=3% is that there is no obvious trend in wall shear stress reduction with increasing Reynolds number. Rather, the values fluctuate around $(c_{f}/c_{f,0\%})=0.90\pm 0.03$.

### 3.2. Global Influence of Particle Migration on the Flow Losses in the Channel

**Figure 11:**
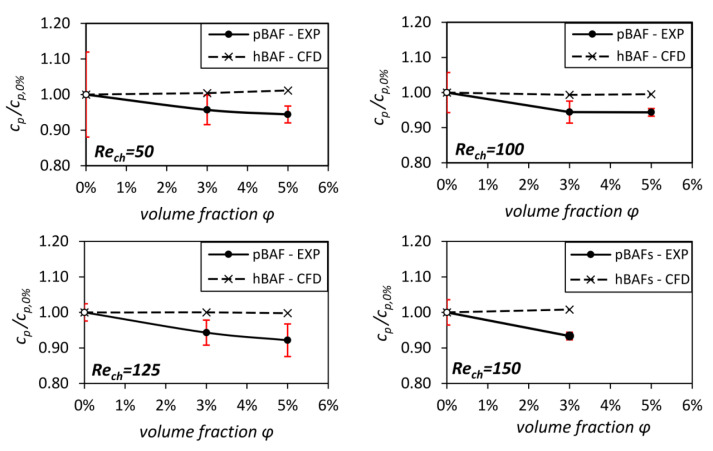
Pressure loss coefficients $c_{p}$ in the microchannels at different volume fractions and Reynolds numbers. The values were normalized with the respective 0% value (white dots). The red lines indicate the measurement uncertainties. Please be aware that CFD considers just an hBAF with a similar density and viscosity at a specific volume fraction ϕ.

After characterizing the near-wall flow and CFL of the pBAFs, the global effect of particle migration on the flow losses is discussed. Analyses were performed for all Reynolds numbers and volume fractions, except for $Re_{ch}=150$ and ϕ=5%. At this condition, the high pressure losses of ≥5 bar exceed the measurement range of the sensors in the *narrow channel*.For all other conditions, the experimentally assessed pressure loss values $c_{p}/c_{p,0\%}$ are shown in Figure 11. The $c_{p}$ values are normalized (as was done with the $c_{f}$ values) with their respective 0% value to better compare between different Re and ϕ values. Additionally to the experimental results, the data from the simulations are shown. These CFD results represent $c_{p}$ values of hBAFs with similar densities and viscosities $\mu_{rheo}$ at a certain volume fraction $\mu_{rheo}$ and are intended as a baseline to illustrate the extent to which particle migration reduces flow losses in the channel.

The normalized $c_{p}$ is nearly one for all homogeneous BAFs, as already seen and discussed with the normalized $c_{f}$ values. However, a different picture emerges for the pBAF flows. A reduction in $c_{p}$ compared to the hBAF flow is evident for all Reynolds numbers, with values in the range of $c_{p}/c_{p,0\%}$ = (0.92–0.95). This reduction in flow losses compared to the losses of the hBAFs is attributed to the reduced shear stresses due to CFL formation. This stress reduction was already observed in the wall shear stress measurements (see Table 2).

In addition, two further derivations are noticeable from Figure 11. It can be seen that $c_{p}/c_{p,0\%}$ shows a slightly decreasing tendency with an increasing volume fraction ϕ. This can be explained by the greater difference in local viscosities between pBAF and hBAF in the CFL region with higher volume fractions. In the CFL region, a relatively high $\mu_{rheo}$ is present in the hBAF flow, whilst just a low $\mu_{rheo,0\%}$ (carrier fluid) is present for the pBAF flow in the same region. When $\mu_{rheo}$ increases with an increased volume fraction (see Figure 4), the differences in stresses also increase between pBAF and hBAF (the relation between stress and viscosity can be seen in Equation (1)). This leads to a more pronounced pressure loss reduction for higher volume fractions.

Moreover, no clear trend in the dependence of the pressure losses on Reynolds number is seen in Figure 11, which means that pressure losses relative to hBAF flow losses remain almost the same for a given volume fraction. This is in agreement with the results shown so far. Figure 9 and Figure 10 identified similar CFL heights and local viscosity distributions in the pBAF flows at a given volume fraction for different Reynolds numbers. Furthermore, the normalized wall shear stresses $\left(c_{f}/c_{f,0\%}\right)$ from Table 2 do not reveal a Reynolds-dependent trend.

Based on these pressure loss analyses, the ratio of apparent viscosity to rheometric bulk viscosity $\mu_{app}/\mu_{rheo}$ was determined for all conditions as explained in Section 3.2. The viscosity values are relatively close to each other in the range of $\mu_{app}/\mu_{rheo}\approx$ (0.925–0.945), which corresponds to the range of pressure loss reduction identified from Figure 11. A noticeable decrease in viscosity $\mu_{app}$ can be seen, showing that the pBAF is able to mimic the Fåhræus–Lindqvist effect. To our knowledge, this is the first time that the Fåhræus–Lindqvist effect has been identified and quantified for a particulate blood analog fluid and for these high Reynolds numbers. A comparison with the apparent viscosity of blood under the same experimental conditions will be the next step for our research.

## 4. Summary of the CFL Characteristics of the pBAFs at High Reynolds Numbers

Through combined experimental studies, detailed insights into particle migration and CFL formation at high Reynolds numbers in the order of $Re_{ch}∼100$ were obtained and directly linked to global measurements of shear stress ($c_{f}$) and pressure loss ($c_{p}$) evolutions. At all Reynolds numbers and volume fractions, CFL formation is observed, which leads to a reduction in shear stresses and to the Fåhræus–Lindqvist effect in the microgeometry.

Furthermore, APTV measurements revealed that an increase in particle volume fraction from ϕ=0.006% to 3% or 5% only induces a very weak decrease in the CFL height of less then 5μm. No significant changes could be observed in CFL height between ϕ=3% an 5%. Complementary measurements at same Reynolds numbers and particle volume fraction revealed a relative pressure loss reduction $c_{p}/c_{p,0\%}$ with the pBAF flows. This can be understood from the following considerations in Equations (3) and (7): the bulk velocity $c_{b}$ increases linearly with $\mu_{rheo}$ to maintain the same Reynolds number (see Equation (3)) for different volume fractions. If the volume fraction is homogeneous, i.e., $\mu_{loc}=\mu_{rheo}$, at any position in the channel, one would expect $\left(p_{f_{2}}-p_{f_{1}}\right)$ to increase likewise such that $c_{p}$ (Equation (7)) remains the same for different volume fractions (as it is with the hBAFs). However, the measured pressure drop $\left(p_{f_{2}}-p_{f_{1}}\right)$ is weaker compared to that of a hBAF flow as a CFL is formed and the near-wall stresses are reduced.

Due to these relative pressure loss changes, the apparent viscosity $\mu_{app}$ is also reduced compared to the measured viscosity $\mu_{rheo}$ from the rheometer. A more dominant decrease in apparent viscosity $\mu_{app}$ compared to $\mu_{rheo}$ is expected when the particle volume fraction (hematocrit) is further increased [19]. For this reason, investigations in the direction of physiological volume fractions (hematocrits) are planned for future work.

The most striking result of this study is that no changes in the CFL height, particle distribution and hence local viscosity distribution are measured, even though the channel Reynolds number triples (here from $Re_{ch}$ = 50 to 150). This trend is confirmed by global measurements of the wall shear stress and pressure loss, where deducted normalized friction coefficients ($c_{f}/c_{f,0\%}$) and pressure loss coefficients ($c_{p}/c_{p,0\%}$) change to similar extents within their measurement uncertainties for all investigated Reynolds numbers. Such a Reynolds number insensitivity was also observed by Matas et al. for suspension flows in tubes and microchannels [52,53].

We assume that the pBAFs are insensitive to the investigated Reynolds number variations, as inertial migration forces dominate the particle migration dynamics and the wall distance at which shear gradient and the wall lift force cancel out (PSSA position) is hardly effected. For a particle system with dominating inertial forces, the CFL is naturally confined by the PSSA position and therefore will also undergo very little changes. How far this trend persists for higher volume fractions, where shear-induced particle migration is expected to also play a major role, is still to be investigated.

A similar trend of Reynolds-independent CFL heights was observed by Stergiou et al. [9] at reduced volume fractions of ϕ=10%. Here, CFL heights reached an asymptotic value with increasing $Re_{ch}$. Their analyses were performed at much lower Reynolds numbers ($Re_{ch}\leq 6$), where migration effects are overall weaker. This suggests that for blood flows with low hematocrits, the migration dynamics are also much more insensitive to Reynolds number variations compared to blood systems with higher hematocrits due to strongly reduced particle interactions.

Based on our results, future investigations aim to directly compare the CFL characteristics of real blood flows and the pBAF flows at hematocrits, particle volume fractions of 5% and higher volume fractions up to physiological values to unravel the sensitivity of the CFL on the channel Reynolds number more in detail.

## 5. Significance of the Results for the Understanding of the Shear Stress Distribution in VAD Gaps

With the results of this study, initial assumptions can be made about the real shear stress distribution in the gap flows of a VAD. As can be seen from the measured results of pressure losses and wall shear stresses, the stresses are reduced in the microgemoetry with the pBAFs compared to the hBAFs for all conditions. For all investigated Reynolds numbers, a wall shear stress is established which is about (10±3)% smaller than the WSS of the equivalent homogeneous fluid, see Table 3. Furthermore, the trends in Figure 11 and Table 2 indicate that as the volume fraction increases, the stresses are further reduced compared to the corresponding flow of hBAF. Therefore, it is hypothesized by us that the effect of stress reduction due to CFL formation is even more pronounced in a gap flow at higher volume fractions, e.g., at ϕ = (35–45)%, which is the physiological hematocrit in our body circulation. We will investigate this hypothesis further in future experiments by increasing the volume fraction in the microchannel to physiological values.

However, this effect of stress reduction is not considered in the current analysis of VAD flow fields, e.g., by numerical flow simulations in VADs. In these simulations, blood fluid modeling assumes a hBAF flow almost exclusively. The results of this study are an indication that numerical fluid modeling should also account for cell migration in narrow VAD gaps. Otherwise, there will be a significant overestimation of stresses in the VAD flow simulation. Further experimental studies, with pBAFs as well as with blood at various hematocrits, will be performed by us in the future to develop a numerical blood flow model that accounts for these migration effects.

## 6. Conclusions

The objective of this paper was to investigate particle migration and its impact on the stress field of blood analog fluids in microfluidic flows at high Reynolds numbers (Re∼100). Similar Reynolds numbers and flow conditions can be found in narrow gaps of ventricular assist devices. In these VAD gaps, high stresses of several hundred Pascals prevail, which might harm the form and function of red blood cells. Hence, an understanding of cell migration and its effect on the stress field is of great interest for this region.

Particle migration was studied in microchannels of 150 microns in height at particle volume fractions up to ϕ=5%. It was found that a cell-free layer (CFL) is formed under all conditions, which indicates Reynolds number independence at a given volume fraction ϕ. Particle migration and CFL formation lead to a locally inhomogeneous viscosity distribution (Figure 10), which reduces shear stresses in the microchannel significantly, as indicated by wall shear stress (Table 2 and Table 3) and pressure drop measurements (Figure 11). The pressure measurements also allowed the determination of apparent viscosity, which showed that the Fåhræus–Lindqvist effect occurs in the studied flow.

If the described flow conditions also occur in VAD gaps, this would imply that the stress field acting on the blood cells in the gap is strongly dependent on cell migration. Due to this migration, lower stresses would act on the cells in the VAD gap than previously thought.

However, further studies are needed to verify this. To date, only particulate blood analog fluids have been used at low hematocrits/volume fractions. Further studies with animal blood and higher hematocrits (ϕ≈30%) are needed to investigate cell migration under physiological conditions. Here, it is particularly interesting to understand the forces acting on the cells/particles at higher volume fractions and higher Reynolds numbers. Another point is to study the effect of differences in migration and forces on the rigid particles in the pBAF and the deformable RBCs in blood. Moreover, these studies were only performed in microchannels with flow conditions similar to those in VAD gaps. In the future, more application-oriented studies of cell/particle migration in the real gaps of VADs are needed to show that the effects observed in this work also prevail in the VAD gap. These studies will also be performed by us in the future.

## Figures and Tables

**Table 1:** Composition of the working fluids. hBAF denotes a (particle-free) blood analog fluid with homogeneous fluid properties. pBAF is an acronym for a particulate blood analog fluid, where heterogeneous fluid properties exist due to heterogeneous particle distributions.

Acronym	Carrier Fluid	Particle Volume Fraction ϕ	Density ρ
RIM-0 (hBAF)	water–glycerol–ammonium thiocyanate	0.006% (v/v) PMMA	1178 kg/m${}^{3}$
RIM-3 (pBAF)		3% (v/v) PMMA	1178 kg/m${}^{3}$
RIM-5 (pBAF)		5% (v/v) PMMA	1179 kg/m${}^{3}$
PEG-0 (hBAF)	water–polyethylene glycol 200	0% (v/v) PS	1047 kg/m${}^{3}$
PEG-3 (pBAF)		3% (v/v) PS	1054 kg/m${}^{3}$
PEG-5 (pBAF)		5% (v/v) PS	1065 kg/m${}^{3}$

**Table 2:** Friction coefficient $c_{f}$ representing the wall shear stresses of pBAFs and hBAFs at different $Re_{ch}$ and volume fractions ϕ. The friction coefficients were normalized by their fraction-specific 0% value for better comparability between all conditions. Please be aware that the CFD considers just an hBAF with a similar density and bulk viscosity at a specific volume fraction.

		Normalized Friction Coefficient $c_{f}/c_{f,0\%}$		
Reynolds Number	Fluid (Method)	ϕ=0%	ϕ=3%	ϕ=5%
$Re_{ch}=75$	hBAF (CFD)	1	1	1
	pBAF (EXP)	1	**0.90** (−10%)	**0.87** (−13%)
$Re_{ch}=100$	hBAF (CFD)	1	1	-
	pBAF (EXP)	1	**0.88** (−12%)	-
$Re_{ch}=125$	hBAF (CFD)	1	1	-
	pBAF (EXP)	1	**0.93** (−7%)	-
$Re_{ch}=150$	hBAF (CFD)	1	1	-
	pBAF (EXP)	1	**0.90** (−10%)	-

**Table 3:** Absolute WSS values for ϕ=0% and ϕ=3% for three different Reynolds numbers. These results are the dimensional results of Table 2.

		Absolute WSS Value $\tau_{w}$	
Reynolds Number	Fluid (Method)	ϕ=0%	ϕ=3%
$Re_{ch}=100$	hBAF (CFD)	105 Pa	189 Pa
	pBAF (EXP)	105 Pa	167 Pa (−12%)
$Re_{ch}=125$	hBAF (CFD)	131 Pa	237 Pa
	pBAF (EXP)	131 Pa	219 Pa (−7%)
$Re_{ch}=150$	hBAF (CFD)	157 Pa	283 Pa
	pBAF (EXP)	159 Pa	257 Pa (−10%)

## Data Availability

Not applicable.

## Supplementary Materials

The following supporting information can be downloaded at: https://www.mdpi.com/article/10.3390/mi14081494/s1, Figure S1: Visualization of the single steps for the analysis of the local particle distribution in the microchannel by APTV.

## Nomenclature and Abbreviations

The following nomenclature are used in this manuscript:

$c_{b}$	Bulk Velocity	(m/s)
$c_{f}$	Skin Friction Coefficient	(-)
$c_{p}$	Pressure Coefficient	(-)
$D_{p}$	Particle Diameter	(m)
$D_{h}$	Hydraulic Diameter	(m)
*h*	Channel Height	(m)
*L*	Channel Length	(m)
$Re_{ch}$	Channel Reynolds Number	(-)
$Re_{p}$	Particle Reynolds Number	(-)
$u_{i},u_{j}$	Velocities	(m/s)
*w*	Channel Width	(m)
$x_{i},x_{j}$	Spatial Directions	(m)
$\mu_{app}$	Apparent Viscosity	(mPas)
$\mu_{loc}$	Local Viscosity	(mPas)
$\mu_{rheo}$	Bulk Viscosity (from the Rheometer)	(mPas)
ρ	Density	(kg/m${}^{3}$)
$\tau_{ij}$	Shear Stress	(Pa)
$\tau_{w}$	Wall Shear Stress	(Pa)
ϕ	Particle Volume Fraction	(%)
$\phi_{loc}$	Local Particle Distribution	(-)

The following abbreviations are used in this manuscript:

APTV	Astigmatism Particle Tracking Velocimetry
BAF	Blood Analog Fluid
hBAF	Homogeneous Blood Analog Fluid
pBAF	Particulate Blood Analog Fluid
CFD	Computational Fluid Dynamics
CFL	Cell-Free Layer
DNS	Direct Numerical Simulation
EXP	Experiment
PMMA	Polymethylmethacrylat
PS	Polystyrol
PSSA	Pseudo Serge Silberberg Annulus
RIM	Refractive Index Matched
RBC	Red Blood Cell
VAD	Ventricular Assist Device
WSS	Wall Shear Stress
