# Supplementary material for: Effect of Particle Migration on the Stress Field in Microfluidic Flows of Blood Analog Fluids at High Reynolds Numbers
Source: Micromachines (Basel). 2023 Jul 25;14(8):1494. doi: 10.3390/mi14081494 (PMC10456677; doi:10.3390/mi14081494)
Supplement: Supplementary file 1 [file micromachines-14-01494-s001.zip › micromachines-2514158-supplementary.pdf]

Supplementary Materials

Figure S1 shows the processing steps to determine the local particle distribution, which is visualized in Figure 9 in the manuscript. APTV is a single-camera technique allowing to extend a planar image-based 2D-PTV approach to a 3D tracking approach with the help of astigmatic particle images. In the first step, a real-time image is recorded with the CCD camera from the top view. Using a predefined calibration curve, an exact height position in the microchannel (h-position) can be determined depending on the fluorescence of each particle. After this step, the counted particles can be positioned in the channel (Step 3). In a further processing step, the local particle distribution can be determined for all the particles at the respective height.

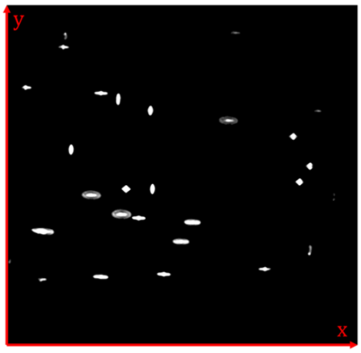

***Step 1**: A real-time image captured by the CCD camera in APTV measurement*

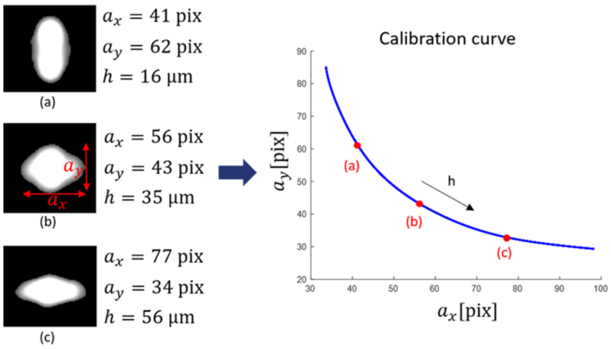

***Step 2**: A calibration curve and the individual particle images with different aspect ratios as an example*

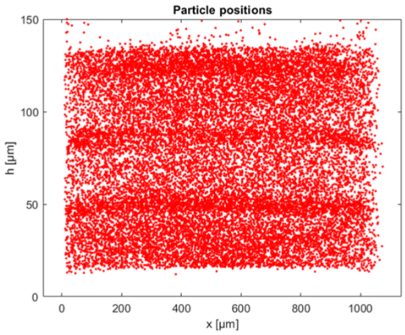

***Step 3**: Particle positions after APTV analysis of h-x plane where x is a part of the channel length*

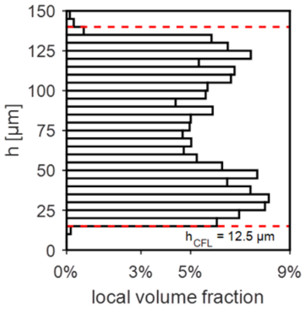

***Step 4**: Particle distribution in the microchannel obtained from the analysis of all particle positions*

Figure S1. Visualization of the single steps for the analysis of the local particle distribution in the microchannel by APTV.
